# Digital phenotyping of depression during pregnancy using self-report data

**DOI:** 10.1016/j.jad.2024.08.029

**Published:** 2024-08-11

**Authors:** Kristen Allen, Samantha Rodriguez, Laila Hayani, Scott Rothenberger, Eydie Moses-Kolko, Hyagriv N. Simhan, Tamar Krishnamurti

**Affiliations:** a Department of Engineering and Public Policy, Carnegie Mellon University, Pittsburgh, PA, United States of America; b Allegheny County Department of Human Services, Pittsburgh, PA, United States of America; c Division of General Internal Medicine, University of Pittsburgh, Pittsburgh, PA, United States of America; d Naima Health LLC, Pittsburgh, PA, United States of America; e University of Pittsburgh Medical Center Western Psychiatric Hospital, Pittsburgh, PA, United States of America; f Department of OB-GYN and Reproductive Sciences, University of Pittsburgh, Pittsburgh, PA, United States of America

**Keywords:** Depression, Pregnancy, Mhealth, Risk modeling, Natural language processing, Machine learning

## Abstract

**Background:**

Depression is a common pregnancy complication yet is often under-detected and, subsequently, undertreated. Data collected through mobile health tools may be used to support the identification of depression symptoms in pregnancy.

**Methods:**

An observational cohort study of 2062 pregnancies collected self-reports of patient history, mood, pregnancy-specific symptoms, and written language using a prenatal support app. These app inputs were used to model depression risk in subsequent 30- and 60-day periods throughout pregnancy. A selective inference lasso modeling approach examined the individual and additive value of each type of patient-reported app input.

**Results:**

Depression models ranged in predictive power (AUC value of 0.64–0.83), depending on the type of inputs. The most predictive model included personal history, daily mood, and acute pregnancy-related symptoms (e.g., severe vomiting, cramping). Across models, daily mood was the strongest indicator of depression symptoms in the following month. Models that retained natural language inputs typically improved predictive accuracy and offered insight into the lived context associated with experiencing depression.

**Limitations:**

Our findings are not generalizable beyond a digitally literate patient population that is self-motivated to report data during pregnancy.

**Conclusions:**

Simple patient reported data, including sparse language, shared directly via digital tools may support earlier depression symptom identification and a more nuanced understanding of depression context.

## 1. Introduction

Perinatal depression is associated with poor maternal and infant wellbeing. Maternal depression has been linked to adverse birth outcomes, such as preterm birth and low birth weights (Chung et al., 2001; Grote et al., 2010; Jarde et al., 2016) as well as delayed infant development (Black et al., 2017; Cummings and Davies, 1994; Cummings et al., 2008; Elgar et al., 2007; Field, 2011; Grace et al., 2003; Lim et al., 2008; Lovejoy et al., 2000). Maternal suicide is a leading cause of postpartum mortality (Campbell et al., 2021; Lindahl et al., 2005).

During pregnancy, approximately 20 % of women have documented depression (Yin et al., 2021). Experienced depression rates in pregnancy may be even higher due to social stigma surrounding disclosure and constraints on the time available in prenatal visits to routinely address mental health (Dennis and Chung-Lee, 2006). While validated screening instruments for perinatal depression are available (e.g., Edinburgh Postnatal Depression Scale (Cox et al., 1987) and PHQ-9/PHQ-2 (Kroenke et al., 2001)), they are not administered consistently during prenatal care (Sidebottom et al., 2021), resulting in lack of adequate detection or referral to recommended care. Moreover, while these measures capture severity of depression symptoms, which are highly predictive of psychiatric diagnosis, they offer limited insight into the underlying context in which the symptoms are being experienced.

Digital health apps have become ubiquitous in United States healthcare delivery (Abernethy et al., 2022), with more mobile health apps available for use in pregnancy than for any other medical condition (Hughson et al., 2018). Tools designed or delivered by healthcare systems offer an opportunity to foster ongoing connection to a healthcare provider, particularly during the time in between routine care (de Mooij et al., 2018). Many individuals report a preference to use smartphones to disclose and receive feedback about personal or sensitive health information (Krebs and Duncan, 2015). Therefore, smartphone apps may also offer a way for pregnant people to disclose stigmatized information more easily (Krishnamurti et al., 2021). App data has been used to successfully provide insights into mental health state (Prakash et al., 2021; Torous et al., 2016; Torous et al., 2015). Such digital phenotyping, in which active data from the patient, passive data (e.g., from sensors), and even digital biomarkers, such as scrolling and tapping behavior, shows promise for personalizing mental health care (Prakash et al., 2021). Increasingly, mobile health technologies are being used to support perinatal mood disorders, yet the data on their use for mood disorder prediction or symptom monitoring is more limited (Novick et al., 2022). Here, we examine several forms of inputs into a prenatal health app to understand their value in predicting and explaining depression risk in a pregnant population.

Using an app prescribed by obstetrical care providers to their patients for general use during pregnancy, we collected patient history, daily mood, and a range of pregnancy-related symptoms through app-embedded survey functions. First person narrative text entries were collected in response to open-ended prompts. Self-completed depression screeners were available at all times and explicitly prompted in each pregnancy trimester. We sought to understand the value of this digital self-report data in modeling depression symptoms during pregnancy.

## 2. Methods

### 2.1. Participant enrollment

The MyHealthyPregnancy smartphone application (Apple version 1.4.7 or Android version 1.8) was prescribed to patients receiving prenatal care in the University of Pittsburgh Medical Center healthcare system as part of a quality improvement initiative (Quality Improvement/Ethics board approval project number: 1684). This app was prescribed across the pregnant population, typically at the first prenatal visit, as a means to offer pregnancy risk identification and support. The app included educational content tailored to the individual user based on their clinical characteristics and gestational week, provided a fetal movement counter and contraction counter, and offered opportunities to complete routine psychosocial risk screenings and document other aspects of their pregnancy experience. The app offered recommended resources in response to the data entered (e.g., connection to social support groups or classes) or provided recommended actions (e.g., prompts to call 911 or the individual’s prenatal care provider or to engage in watchful waiting). Select data on specific risk factors identified through the app (e.g., reported decreased fetal movement) were transferred to a portal integrated with the patient’s medical record for access by their prenatal care provider. Any individuals reporting moderate-to-severe depression symptoms or suicidal ideation were reached out to by a member of a dedicated nursing team who provided the appropriate resources, care, or referral (e.g., perinatal psychiatric or other behavioral health services). The app’s content was developed in partnership with a clinical education team employed by the healthcare system. Upon downloading the app and creating an account, participants electronically consented to sharing identifiable data with their healthcare provider and to dissemination of de-identified aggregate data for scientific research. This secondary data analysis was approved by the University of Pittsburgh’s Institutional Review Board (STUDY19100210). Financial compensation was not provided to patients for app use.

### 2.2. Data collection

Upon initiation of app use, participants completed baseline questions about their pregnancy-relevant personal history, including demographic information and history of diagnosed depressive and anxiety disorders. Participants could record information about their pregnancy experience on a routine (up to daily) basis using a “check-in” questionnaire. This questionnaire included a one-dimensional ecological momentary assessment question about mood, designed for brevity and simplicity, in which participants responded to “How is your mood today?” with a 5-point Likert scale structure common among such affect and wellbeing measures (Asselbergs et al., 2016; Salsman et al., 2014) ranging from 1 (“Very poor”) to 5 (“Very good”). Participants could also select any physical symptoms they were currently experiencing from a pre-populated list provided to them as part of the daily questionnaire. If they were experiencing a symptom that was not included in the pre-specified list, they could select “other symptom, not listed” and manually enter a description of their symptom.

Throughout their pregnancy, participants were also able to share open-ended text in the app in several ways. The app contained a “journaling/notes” section where app-users could choose to document thoughts, feelings, symptoms, or other notes. App users also had the opportunity to provide responses to open-ended questions asked on either a weekly or monthly basis about, for example, their current relationships or their mood that day (e.g., *What had the biggest impact on your mood today, and why?*). A COVID symptom screener, deployed through the app from April 2020 to September 2022, also asked open-ended questions about COVID-19 information sources, preferred methods to prevent infection, and pandemic-related challenges (e.g., *Are you experiencing financial or other personal difficulties as a result of this pandemic?*).

Lastly, the app provided a voluntary opportunity to complete the Edinburgh Postnatal Depression Scale (EPDS) by prompting them on the app’s home screen to complete it once a trimester. This depression risk measure was selected as it represented the standard of care for peripartum mental health screening in the system in which the app was deployed.

### 2.3. Inclusion criteria

All users were eligible to participate if they provided consent for analysis of data for research purposes, completed baseline demographic and personal medical history information, completed an EPDS at least once, reported on daily mood at least once, and entered at least one or more usable open-ended text entry within a 60-day window prior to providing an EPDS score. Usable text entries were defined as English-language entries that retained at least one word after concatenated text entries had been processed to remove less meaningful words (e.g. “I”, “be”, and “did”) and overly common single-response words (e.g. “yes”). More detail describing these text entries is discussed in a prior publication on a subset of this data (Krishnamurti et al., 2022a).

### 2.4. Data structuring

After responding to all questions from the in-app EPDS, a score of at least 14, or a score < 14 paired with reports of suicidal ideation, flagged a user as experiencing moderate-to-severe depressive symptoms. This scoring approach is the recommended threshold for major depression during pregnancy (Murray and Cox, 1990) and within the range of validated EPDS scoring thresholds for moderate-to-severe depression (Hewitt et al., 2009; Levis et al., 2020). It was selected for consistency with the healthcare system in which the app was deployed. For inclusion in modeling, input features were implemented as categorical values. Demographic information included the participant’s age at the start of their pregnancy; their race/ethnicity, categorized into white, Black, Hispanic/Latinx, Asian, or other; household income, categorized as a binary variable of less than $50,000 or $50,000+; and education, categorized as a binary variable of less than an associate’s degree or an associate’s degree or higher. Clinical history included self-reported history of depression and self-reported history of anxiety. Mood was categorized into the self-reported minimum, maximum, and average daily mood. Pregnancy-related symptoms were operationalized using four distinct binary features for *critical* (e.g., leaking fluid, fever, moderate-to-severe cramping, etc.), *significant* (e.g., frequent dizziness, swelling in face, vision problems, etc.), *common* (e.g., fatigue, heartburn, nausea, etc.), and *open-ended symptoms* (e.g., hot flashes, back pain).

### 2.5. Natural language feature extraction

Reflecting the two windows often used by clinicians to identify depressive symptoms for a new depression diagnosis, a person’s text entries from 30-day and 60-day periods preceding each instance of EPDS measurement were matched with the self-report score.

Text data was first processed by grouping together open-ended entries with EPDS scores. Text entries that were provided in close temporal proximity (e.g., a journal entry on Day 1 and an open-ended prompt response on Day 10) were concatenated. Concatenated text occurring in a 30/60-day period was then paired with the average of all following EPDS scores within the 30/60 days from the last open-ended text entry, using only EPDS scores before the occurrence of a newer text entry. Within both the 30- and 60-day timeframes, EPDS scores with no preceding text entries and text entries with no following EPDS scores were eliminated from the dataset.

Several established natural language processing approaches, described below, were then applied to the texts to extract language features.

### 2.6. Sentiment analysis

Sentiment analysis tools estimate positive or negative valence in words and provide clues to the writer’s attitude about their subject (Baccianella et al., 2010). Here, text *sentiment* was analyzed using SentiWordNet (Baccianella et al., 2010). Each word extracted from a processed text entry was given both a positive and a negative score using a stemmed SentiWordNet dictionary. Positive and negative scores across a concatenated piece of text were aggregated to give an average positive and negative sentiment. Overall fluctuation in sentiment was calculated by subtracting the average negative from average positive scores, yielding a single value representing the breadth of emotional content present in a period of writing.

### 2.7. Topics/themes

Topic modeling can elucidate emergent themes particular to specific domains of writing (Blei et al., 2003) that may not be present in more generalized language datasets developed on news articles and historical texts. Here, *topic modeling* was performed using Latent Dirichlet Allocation (LDA), an unsupervised machine learning method that clusters data points into a specified number of topics (Blei et al., 2003). Topic models permit us to examine domain-specific patterns that may emerge in this collection of text by pregnant people by illustrating which less-common words frequently occur together in this body of text.

The Linguistic Inquiry and Word Count dictionary (LIWC-22), where expert insight seeded psychologically-informed themes, has shown utility in identifying pre-specified topics associated with mental health issues (Boyd et al., 2022; Coppersmith et al., 2014; Zirikly et al., 2019). LIWC-22 was used to count the number of occurrences of 119 themes, grammatical features, and positive and negative affect within each text entry (Boyd et al., 2022). In addition to 117 LIWC-22 themes, two additional themes—COVID-19 and pregnancy-terms—were manually created to capture domain-specific content. The COVID-19 theme included terminology related to the global health crisis, such as “mask”, “booster”, and “pandemic”. The pregnancy-terms theme captured pregnancy terminology not fully captured in the LIWC health category (e.g., “contractions,” “doula”).

### 2.8. Semantic/syntactic structure

Word embeddings incorporate high-dimensional context-derived representations of *syntactic and semantic information* for each concatenated text entry. The 300-dimensional word2vec embeddings were pretrained on word co-occurrence and proximity in 1.6 million news articles, assigning similar representations to words that usually showed up in the same contexts (Mikolov et al., 2013a). While the approach produces systematic regularity among word embeddings that captures meaningful information about semantic and syntactic roles (Mikolov et al., 2013b), these embeddings are not intuitively interpretable. We implemented a matrix rotation algorithm (Park et al., 2017) that encourages the word embedding matrix to reduce to only a few large values both row-wise and column-wise, reducing overlap between embeddings and increasing interpretability. Applying the rotation algorithm preserves the semantic and syntactic information from the original embeddings, as demonstrated by comparable predictive accuracy across a variety of classic language processing tasks (Park et al., 2017), and results in more distinct language characteristics being apparent within each vector.

### 2.9. Statistical analyses

EPDS scores were predicted for each data point in the data set using a stepwise regression strategy (Beale et al., 1967; Efroymson, 1960), whereby the additive value of each type of input - personal history (demographic and clinical history information), mood, pregnancy-related symptoms, and extracted natural language features - was examined alone and in combination with each and all of the other input types in a series of lasso models. To increase the inferential strength of the lasso-selected coefficients, we applied exact post-selection inference (SI) (Lee et al., 2016) for lasso (Taylor and Tibshirani, 2018) which assumes lasso correctly selected a superset of relevant variables, but that the coefficient values are not robust enough for inference and generalizability. Coefficient selection by traditional lasso implies a given model must either a) be considered relevant only for the study-specific population and not generalizable or b) subject to stronger significance criteria (Taylor and Tibshirani, 2015). SI lasso searches for the optimal linear combination of coefficients that minimizes the expected error of predictions, resulting in clear coefficient confidence intervals and more generalizable interpretations. Sample code outlining the SI lasso step-wise strategy can be found in supplemental materials (Supplemental Code). All regression models were run on input occurring in a 30-day and a 60-day timeframe before EPDS response.

## 3. Results

### 3.1. Study participants

**Fig. 1.**
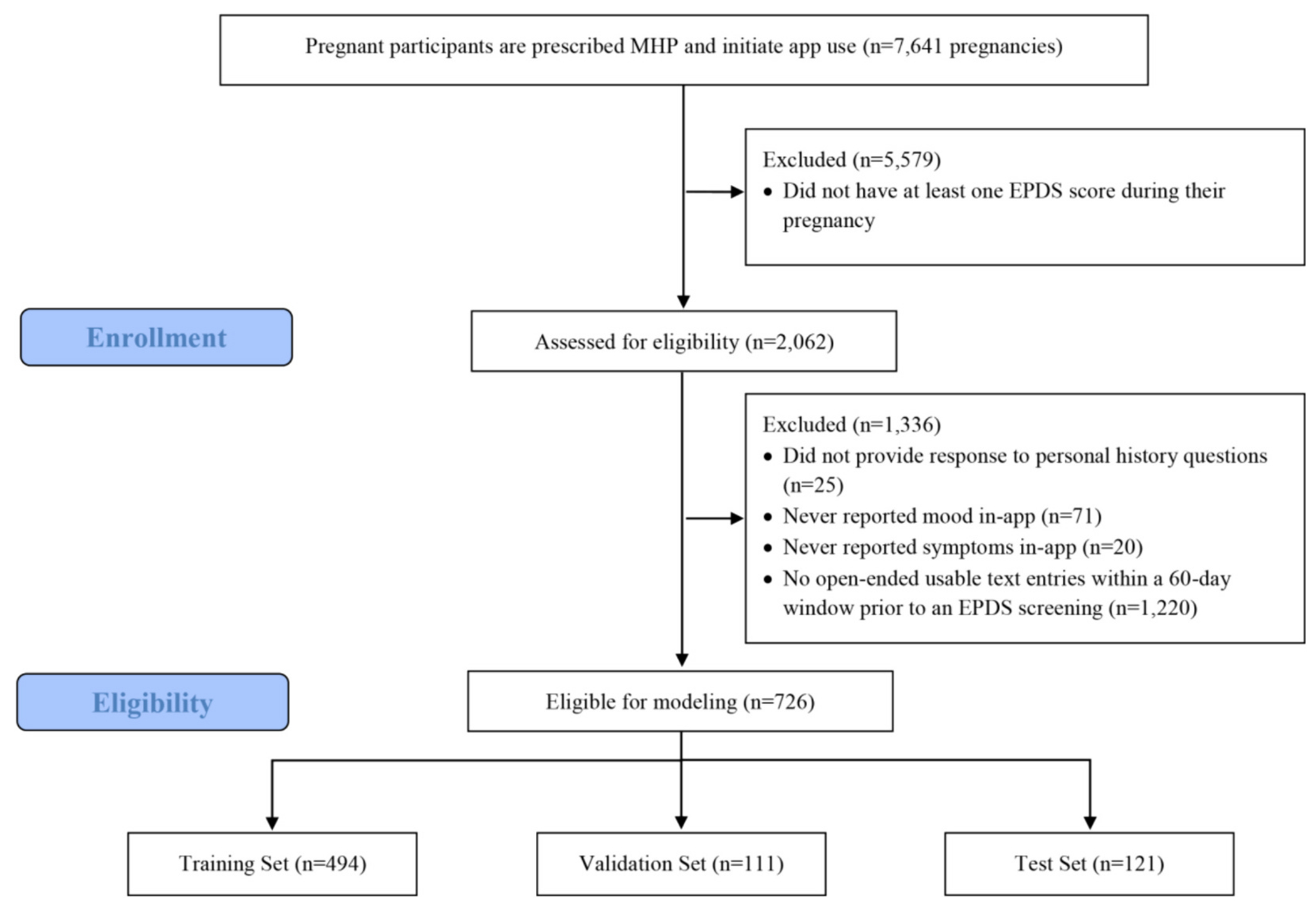
STROBE flow diagram. Flowchart of enrollment in study, eligibility criteria for modeling, and split into training, validation, and testing groups.

During the study period of September 2019 – October 2022, the MyHealthyPregnancy^™^ app was used by 7455 individuals who had been prescribed the app as part of their routine prenatal care. Of those individuals, a total of 726 pregnancies provided complete data for analysis. This subset of app users was then randomly divided into a *training set* (70 %), which we used to identify model coefficients, a *validation set* (15 %), which informed the model selected for the *training set*, and a *test set* (15 %), to evaluate the final model. Fig. 1 shows participant flow through the study.

The demographic characteristics of those eligible for inclusion in the study were largely reflective of the greater population of MyHealthyPregnancy app users (Table 1). Most participants identified as non-Hispanic whites and were partnered. The mean age of participants was 29.5 years (SD = 5.5 years). Most had a college education or higher, and the majority reported a family household income of at least $50,000 annually. Participants who provided sufficient data for study inclusion were more likely to have a household income of less than $50,000/year, were more likely to report histories of depression and/or anxiety, more often nulliparous, less likely to identify as Asian, and less likely to have a college degree (Supplemental Table 1).

### 3.2. Depression screening

Participants completed an EPDS on average 2.5 (SD = 2.3) times over the course of their pregnancy, with the median time of first completion in their 11th week of pregnancy. Among all averaged EPDS scores that were eligible for modeling, 55.0 % indicated few to no depression symptoms (score of 0–6), 34.6 % indicated mild depression symptoms (score of 7–13), 7.7 % indicated moderate depression symptoms (score of 14–19), and 1.9 % indicated severe depression symptoms (score of 20–30). These EPDS scoring cut-points reflect those used by the healthcare system where the work was completed. One percent (17/1496) of EPDS scores included in modeling indicated suicidal ideation.

### 3.3. Mood and symptoms

Participants provided an average (SD) of 5.77 (10.26) mood self-reports, with an average mean mood per participant of 3.87 (0.69) on a 5-point Likert scale, an average minimum mood of 3.45 (1.01), and an average maximum mood of 4.17 (0.71). Symptoms reported by study-eligible individuals fell into three categories of pre-specified pregnancy-related symptoms– *critical symptoms*, associated with an emergent health issue (*n* = 413 reports from 270 participants, representing a total of 1332 symptom reports), *significant symptoms*, warranting discussion with a provider (*n* = 603 reports from 359 participants, representing 4426 total symptom reports), and *common symptoms* associated with the normal trajectory of pregnancy (*n* = 1020 reports from 634 participants, representing 29,572 total symptom reports), as well as *open-ended symptoms* entered by participants that were not in a prespecified list (*n* = 303 reports from 183 users, representing 903 total symptom reports).

### 3.4. Open-ended text entries

Participants provided open-ended text through different features of the tool, including responses to specific prompts and a pregnancy journaling section of the app. Included participants had an average (SD) of 8 (16) text entries, with an average length of 19 (SD = 28) words. EPDS scores did not significantly differ between participants who submitted open-ended text and those who did not, *χ*^2^ (26, *N* = 1227) = 31.6, *p* = .21. However, there was a significant positive correlation between text lengths and depression symptom score, *r*(764) = 0.10, *p* < .01.

Lengthier entries often included descriptions of multiple experiences, such as physical symptoms, notes from medical appointments, and narratives of personal events. A participant might share a journal entry such as,

“I’m obsessing, getting the baby’s room ready. There’s a ton of stuff out there but not sure what’s immediately necessary. Also had a migraine this morning (took some meds) and issues with food aversions this week. Having some mood swings too, it comes and goes throughout the day but it’s alright for now.”

Shorter entries were typically explanations in response to open-ended prompts (e.g., “*What had the biggest impact on your mood today, and why?*” with a sample response of “*Good mood today b/c of ultrasound*”) rather than unprompted entries.

### 3.5. Depression risk models

Table 2 shows the full set of models of moderate-to-severe EPDS scores in a 30-day period.

In the 30-day timeframe, the 15 models shown in Table 2 range in test AUC from 0.69 to 0.83, selecting at most 7 features from the larger set of 445 potential features. Six models have an AUC value of >0.80. The top-performing models incorporate the individual’s personal clinical history and mood (Table 2, Model 8; Model 14). Across models, with the exception of one (Table 2, Model 13), the addition of mood into a model improves the model’s AUC. The distinct addition of personal history or pregnancy-related symptoms also typically maintains or improves model performance.

Several language features were retained in the six highest performing models. Specifically, a more negative writing *tone*, a LIWC feature that represents the sum of positively coded words (e.g., beauty, calm, optimism) and negatively coded words (e.g., poor, sad, destructive), is associated with greater depressive symptoms. Conversely, a more positive tone is associated with fewer depression symptoms. Less variation in *sentiment fluctuation* (SentiWordNet differences in positive and negative sentiment scores) is associated with greater depressive symptoms. Use of LIWC *want* words, indicating the writer’s desires through action words, such as *wish*, *hoping*, *craving*, etc. (e.g., “*I’ve been struggling to sleep because of how nauseous I am Hoping it gets better and I have more energy today*.”) was associated with greater depression, perhaps indicating unfulfilled desires. Similarly, word2vec themes, which we labeled as ‘finances,’ captured by writing about topics such as reductions in pay, losing a job, and mortgage/rent payments (e.g., “*I’m starting a new job this week, which is good! But I haven’t told them about the pregnancy yet… definitely worried about them not wanting to hire me*.”) and ‘mental health/health services,’ including discussion of medications and therapy (e.g., “*What if I am one of those moms that resents her baby? I should talk to my therapist about it… Maybe I need meds?*”) are both associated with increased depressive symptoms. No specific themes emerged as protective against depression. A supplemental figure (Supplemental Fig. 1) shows the relative significance level of these features in the top-performing models as well as that of specific mood (average mood), personal history (age, income, and depression/anxiety history), and pregnancy-specific symptom severity (critical and significant). Notably, however, Supplemental Fig. 1 illustrates that among models that include both mood and natural language features as inputs, language features are not retained. This suggests that the predictive value of these language features may be largely, if not fully, captured by mood, while the features themselves offer insight into the underlying drivers of that mood.

The primary value of including natural language in depression modeling, therefore, may be insight into the context of depressive symptoms. A text sample, modified slightly for anonymity and indicative of moderate to severe depression symptoms, follows:

“Not seeing my baby’s dad since Saturday morning was rough. Conflicting work schedules and other family demands and just not having any ‘us’ time. Being alone last night made me pretty emotional. Lots of ups and downs. I only fell asleep when my headache got too bad. I can’t really feel supported because of all the stress. Something’s going on with my mental health. I don’t know whether it has much to do with my medications - or lack thereof - or maybe the absence of counseling but it sucks. It’s like I have absolutely no control over what I’m thinking and I’m worried that my stress could affect the baby. Barely being able to breathe because of things that happened a year ago, even 5 years ago - it’s crippling.”

In contrast, a sample text of an individual with minimal depression risk symptoms is shown below:

“Baby girl is getting really kicky recently. It’s so strange and nice to feel her. And my man has been such a great support during my pregnancy so far. He has pampered me and taken care of me! We laughed today about my nipples, how they look like gumdrops – hahaha. On the plus side, the nausea is starting to dwindle! I treated myself to a Primanti’s sandwich - definitely a Yinzer baby!”

Models for the 60-day period were comparable to those for the 30-day period, but generally showed somewhat less predictive power (see Supplemental Table 2). Only one model (Supplemental Table 2, Model 8), which included personal history and symptoms, showed a slight increase in performance in the 60-day timeframe (AUC = 0.74), as compared to the 30-day timeframe (AUC = 0.72).

## 4. Discussion

In this observational prospective cohort study, we modeled depression symptoms using patient-reported inputs collected through a pregnancy smartphone app that was delivered to patients as part of their routine prenatal care. The study identified distinct ways depression symptoms manifest through patient-reported data, including data that is routinely available to clinical personnel (such as demographic data and medical history), as well as data that is usually not available to clinical personnel but could have value as indicators of mental health, such as mood and natural language use. Our results contribute to a growing body of literature suggesting that patient-reported data, like that collected through mobile health tools, can be used in the prediction of depression symptoms (Andreasen and Pfohl, 1976; Birjali et al., 2017; Bufano et al., 2023; Burkhardt et al., 2021; Chancellor and De Choudhury, 2020; Chiong et al., 2021; Goltermann et al., 2021; Ji et al., 2020; Liu et al., 2022; Moshe et al., 2021; Nguyen et al., 2022; Opoku Asare et al., 2022; Ramos et al., 2019; Tsakalidis et al., 2022; Young et al., 2023). Average mood – rather than the highest or lowest mood measured – was the strongest indicator of depression symptoms, possibly describing the recurring nature of depression symptoms and suggesting that measuring mood alone can be a simple and effective identifier of depression severity. The elements of personal history retained as predictive of depression risk, from among those measured, included younger age, lower household income, and a history of diagnosed depression or anxiety, which are consistent with prior literature (Robertson et al., 2004). The experience of critical pregnancy-related symptoms, as opposed to slightly less serious or even routine pregnancy-related symptoms, was also retained as predictive of depression risk, suggesting that patients who are experiencing acute and serious pregnancy-related symptoms may also warrant mental health support in addition to acute clinical care.

While mood encompassed the predictive value of language, the specific language features retained in models without mood can shed light on the types of topics and valence of language that current mood may be capturing. For example, natural language features that convey emotional valence, namely *tone* and *sentiment fluctuation*, were present across depression prediction models. These findings are consistent with a body of literature suggesting that negative valence is associated with depression risk (Glenn et al., 2018; McCoy Jr et al., 2019).

Additionally, a lack of fluctuation in a text’s sentiment is symptomatic of depression, consistent with findings that language that has less affective variation (i.e., “flat” affect) is associated with depression symptoms (Clark and Watson, 1991; Loveys et al., 2017; Scherer et al., 2015); mirroring the importance of average mood in our own models.

We also find that rotated word2vec embeddings indicated severity of depression symptoms. These embeddings represented the underlying semantic/syntactic structure of the open-ended text provided by patients, including themes of ‘*finances*,’ and ‘*mental health*/*health services*’. That individuals who had moderate or severe depression symptoms wrote about their mental health (e.g., *depression*, *stress*, *psychiatrist*) is suggestive that those writing had an understanding of their depression symptoms and were sensitive to the ebbs and flows of their mental health. Previous studies have shown that individuals with depression may find writing therapeutic (Pennebaker, 1997), while other studies have found that it can be either harmful or therapeutic to re-imagine prior life events (Watkins, 2008). When given the chance to share written thoughts in the pregnancy app, those individuals with symptoms of depression opted to write more extensively than those who were not depressed and to focus on their mental health in that writing. Individuals’ explicit references to mental health services (e.g., *counseling*, *psychiatrist*, *therapist*) could reflect the writer’s self-identification of an unmet need for resources. These findings highlight the value of consistent depression risk screening paired with follow-through treatment throughout pregnancy, as recommended by the American College of Obstetricians and Gynecologists guidelines (ACOG, 2018). Prior work has shown that screening paired with counseling show a 39 % decrease in the likelihood of perinatal depression (U. S. Preventive Services Task Force et al., 2019). Here, it appears that those with a history of depression and at risk of future depression may be fairly aware of that risk. Lastly, the syntactic/semantic theme of ‘*finances*’ and the use of ‘*want*’ words could suggest that, among individuals experiencing physical symptoms, depression severity may correspond less to personal history or a stated need for mental health services than to the financial constraints presented by an acute health issue or the desire to reduce worrisome symptoms.

## 5. Limitations

This study was observational and pragmatic by design as it leveraged an existing digital platform used in clinical practice to support depression risk prediction. As a result, our findings are not generalizable beyond a digitally-literate patient population that is motivated to self-report data during pregnancy. Approximately 96 % of cisgender women of reproductive age in the US own a smartphone (Pew Research Center, 2021) and the majority of pregnant individuals access some sort of perinatal-smartphone app during pregnancy (Lee and Moon, 2016; Mo et al., 2018). However, large surveys show that those with more limited digital access tend to be Black and Brown individuals and beneficiaries of Medicaid insurance (Eyrich et al., 2021; Kan et al., 2022; Roberts and Mehrotra, 2020). These populations also tend to have higher rates of untreated depression (Abrams et al., 2009; Grote et al., 2014; Santoro and Peabody, 2010). Therefore, while using a pregnancy app is one means of collecting self-reported information that is indicative of future depression risk, it certainly should not be used to replace in-person screening or referrals and health equity considerations must be centered in designing such forms of data collection (Krishnamurti et al., 2022b).

Individuals in this study were also self-motivated to share personal, sometimes sensitive information. While there were no demographic differences in those patients who were or were not included in the study, the majority of patients engaging with the MyHealthyPregnancy app did not provide sufficient data to be included in the analyses. This was largely driven by limited sharing of open-ended text data, which is reflective of the challenges documented in other studies soliciting such open-ended text (Ayers et al., 2018; Hayman et al., 2012). This may be because written language is more cumbersome to provide or because it feels more sensitive or personal in nature.

Among those who provided sufficient data, the most common source of writing provided was in response to app-embedded prompts, while the longest entries were from open-ended journals and more likely to come from those with greater depressive symptoms. As a result, the majority of responses are brief and tend to be highly topically focused, while the richest data may overrepresent themes salient to those at greatest risk of depression. It is possible that the number and, especially, the length of the written texts may not have been sufficient to expose more subtle or infrequent, but meaningful themes. This could have limited the degree of usefulness of natural language as an independent predictor of depression risk in our study.

Much of our data collection coincided with the unanticipated COVID-19 pandemic. Periods of mandated self-isolation may have encouraged more writing during this time, making our ability to collect text through the app less generalizable to non-pandemic times. In other work, we documented the increased use of another app-embedded psychosocial screener, which we attribute, in part, to fewer in-person clinical appointments and therefore fewer opportunities to disclose risk directly to a provider (Krishnamurti et al., 2021). Thus, if we ran this study again, we may see less frequent in-app writing or less frequent depression screener completion. It is also possible that depression symptom severity was qualitatively different during this time.

There is evidence that maternal depression risk increased during the height of the pandemic (Bakermans-Kranenburg et al., 2022; Evandrou et al., 2023). However, though COVID-19 was explicitly included as a novel language feature theme, it was not retained as an indicator for depression symptoms in any model. Its omission suggests that other topics that may be pertinent to the pandemic but do not specifically reference COVID-19 precautions or symptoms, such as increased financial constraints and fewer available mental health resources, are more directly indicative of depression. While not the focus of this analysis, data collection from digital tools like this one may offer an opportunity for insights into larger population changes under early stages of sudden social or environmental change.

This is one of the first prospective observational studies of pregnant patients to use self-reported app inputs, including natural language collection (Kelley and Gillan, 2022), to predict depression symptoms. Overall, this research illustrates the potential of using a prenatal care app to collect patient-reported data as a means of both identifying and describing depression symptoms in between routine prenatal care. While the gold standard for depression assessment is a formal clinical diagnosis, our data is limited to using the EPDS score. However, the EPDS has been shown to be highly correlated with clinical diagnosis (Cox et al., 1987; Murray and Cox, 1990; Wang et al., 2021) and is routinely used in clinical care as a proxy for diagnosed depression. While the goal of this work was inference rather than optimized predictive accuracy, the AUCs of our various models was consistent with that of other predictive perinatal depression models in the literature (Cellini et al., 2022), although not as predictive as some designed explicitly for predictive screening purposes (Krishnamurti et al., 2024).

We find that a few self-reported patient measures can predict moderate-to-severe depression symptom onset in the subsequent 30 or 60 days of a pregnancy with a fairly high degree of accuracy (AUC = 0.83). Personal history, mood, and pregnancy-related symptoms all contribute to depression symptom onset, indicating the multi-faceted nature of depression risk and suggesting potential triggers (e.g., low income, pregnancy complications) of depression symptoms. Language inputs alone allow for moderate predictive ability of depressive symptoms among peripartum patients and offer some insights into the context of experienced depression risk factors. In addition to identifying a model for depression risk during pregnancy, this work highlights the potential for using self-report data collected through digital tools to evaluate depression symptoms and to offer supports in between routine prenatal care visits.

## Supplementary Material

Supplementary Material

## Figures and Tables

**Table 1:** Demographics of study participants.

Characteristic	Enrollees (*n* = 2062)
Age, mean (SD)	29.5 (SD = 5.5)
Income, US dollars, thousands	
<15	264 (12.8 %)
15 to <50	505 (24.5 %)
50 to 100	659 (32.0 %)
>100	575 (27.9 %)
Missing or preferred not to respond	59 (2.9 %)
Race and Ethnicity	
White/Caucasian	1640 (79.5 %)
Black or African American	223 (10.8 %)
Hispanic/Latinx	41 (2.0 %)
Asian	71 (3.4 %)
Another ethnicity	77 (3.7 %)
Missing or preferred not to respond	10 (0.5 %)
Educational Level	
<High school	56 (2.7 %)
High school or GED	609 (29.5 %)
Associate degree	236 (11.4 %)
Bachelor degree	585 (28.4 %)
Postgraduate degree	560 (27.2 %)
Missing or preferred not to respond	16 (0.8 %)
Relationship Status	
Partnered	1949 (94.5 %)
Not partnered	107 (5.2 %)
Missing or preferred not to respond	6 (0.3 %)
Medical History	
History of depression only	63 (3.1 %)
History of anxiety only	191 (9.3 %)
History of both depression and anxiety	405 (19.6 %)
No history of depression or anxiety	1403 (68.0 %)
Parity	
Nulliparous	1025 (49.7 %)
Multiparous	1037 (50.3 %)

**Table 2:** The additive effect of personal history, mood, pregnancy-related symptoms, and language features in modeling maternal depression risk.

#	Model description	No. of covariates retained	Test set AUC value [CI]	Test set R^2^
1	Language features	2/429	0.72 [0.60, 0.83]	0.11
2	Personal history	5/9	0.69 [0.57, 0.81]	0.08
3	Mood	2/3	0.78 [0.66, 0.88]	0.19
4	Pregnancy-related symptoms	4/4	0.74 [0.61, 0.85]	0.11
5	Personal history + Language features	6/438	0.72 [0.60, 0.82]	0.11
6	Mood + Language features	1/432	0.78 [0.67, 0.89]	0.19
7	Pregnancy-related symptoms + Language features	5/433	0.81 [0.70, 0.89]	0.18
8	Personal history + Mood	5/12	0.83 [0.73, 0.91]	0.25
9	Personal history + Pregnancy-related symptoms	5/13	0.72 [0.61, 0.83]	0.11
10	Mood + Pregnancy-related symptoms	3/7	0.82 [0.72, 0.91]	0.22
11	Personal history+ Mood + Language features	2/441	0.82 [0.71, 0.91]	0.23
12	Personal history+Pregnancy-related symptoms+ Language features	7/442	0.76 [0.66, 0.85]	0.15
13	Mood + Pregnancy-related symptoms + Language features	1/436	0.78 [0.66, 0.89]	0.19
14	Personal history + Mood + Pregnancy-related symptoms	6/16	0.83 [0.73, 0.91]	0.25
15	Personal history+ Mood + Pregnancy-related symptoms + Language features	4/445	0.82 [0.73, 0.90]	0.23

## Data Availability

Original identifiable data are not publicly available to protect patient privacy and due to the terms and conditions of app data use. Limited de-identified data will be available in the National Institute of Mental Health Data Archives under project 1R21MH119450-01A1.
