# Supplementary material for: Digital phenotyping of depression during pregnancy using self-report data
Source: J Affect Disord. Author manuscript; Available in PMC 2024 Nov 17. (PMC11569620; doi:10.1016/j.jad.2024.08.029)
Supplement: Supplementary Material [file NIHMS2034961-supplement-Supplementary_Material.zip › 1-s2.0-S0165032724012229-mmc3.docx]

**Supplemental Table 1.** **Demographic differences between eligible participants and the general MHP population**

| **Characteristic** | **Patient**  **Cohort (n=7,641)** | **Eligible for Study (n=2,062)** | **Ineligible for Study (n=5,579)** | **P-value^1^** |
| --- | --- | --- | --- | --- |
| Age | 30 (5.29) | 30 (5.47) | 30 (5.22) | .10 |
| Household income above $50,000 | 4,714 (62%) | 1,234 (60%) | 3,480 (62%) | <.01 |
| Associate’s degree or higher | 5,251 (69%) | 1,381 (67%) | 3,870 (69%) | .01 |
| Currently partnered | 7,281 (96%) | 1,949 (95%) | 5,332 (96%) | .10 |
| History of depression | 1,342 (18%) | 468 (23%) | 874 (16%) | <.01 |
| History of anxiety | 1,737 (23%) | 596 (29%) | 1,141 (20%) | <.01 |
| Parity | 3,626 (48%) | 1,025 (50%) | 2,601 (47%) | .02 |
| Race |  |  |  | .04 |
| White/Caucasian | 6,040 (79%) | 1,640 (80%) | 4,400 (79%) |  |
| Black/African American | 796 (10%) | 223 (11%) | 573 (10%) |  |
| Asian | 340 (4%) | 71 (3%) | 269 (5%) |  |
| Hispanic/Latinx | 137 (2%) | 41 (2%) | 96 (2%) |  |
| Other | 266 (3%) | 77 (4%) | 189 (3%) |  |
| Missing | 62 (1%) | 10 (<1%) | 52 (1%) |  |

^1^ p-values compare baseline characteristics between those eligible and ineligible for the study. Age was compared using two-sample t-test, all others compared using χ^2^ test
