# Supplementary material for: Digital phenotyping of depression during pregnancy using self-report data
Source: J Affect Disord. Author manuscript; Available in PMC 2024 Nov 17. (PMC11569620; doi:10.1016/j.jad.2024.08.029)
Supplement: Supplementary Material [file NIHMS2034961-supplement-Supplementary_Material.zip › 1-s2.0-S0165032724012229-mmc1.pdf]

Supplemental Code

Selective inference lasso code sample

The following is a sample of code used to run each selective inference lasso regression. Selective Inference Lasso uses the assumption that lasso will correctly identify a superset of the significant covariates, but in order to make inferential claims, the restrictions on which covariates are truly significant must be bolstered. Note: Any models in this document are specifically within the 30-day time-frame. 60-day time-frame follows the same strategy.

The necessary structure of data used in the below code is as follows:

```
print(dfstructure)
```

```
##   GroupedEPDSAverage traintestdev NLP_var1 OtherNLP_columns PersonlHistory_var1
## 1                  7        train     1.69              ...                 2.7
## 2                 18          dev     1.71              ...               -0.37
## 3                 10        train     1.75              ...               -0.37
## 4                  9         test    -0.54              ...                 2.7
## 5                 20          dev    -0.44              ...               -0.37
## 6                 25        train    -0.67              ...               -0.37
## 7                ...          ...      ...              ...                 ...
##   OtherPersonalHistory_columns Mood_var1 OtherMood_columns
## 1                          ...     -3.25               ...
## 2                          ...      1.11               ...
## 3                          ...     -0.34               ...
## 4                          ...     -0.34               ...
## 5                          ...     -1.79               ...
## 6                          ...      1.11               ...
## 7                          ...      1.11               ...
##   PregnancySymptoms_var1 OtherPregnancySymptoms_columns
## 1                  -0.69                            ...
## 2                  -0.69                            ...
## 3                   1.44                            ...
## 4                  -0.69                            ...
## 5                  -0.69                            ...
## 6                  -0.69                            ...
## 7                   1.44                            ...
```

The below sections of code define the functions used throughout the code. The packages selectiveInference and pROC are required.

```
#AUROC
AUROC_depNLP <- function(probs, yactual){
  positive <- probs[yactual >= 14]
  negative <- probs[yactual < 14]
  score.pairs <- expand.grid(positive = positive,
                             negative = negative)
```

```
  auroc <- (sum(score.pairs$positive>score.pairs$negative)+
              (0.5*sum(score.pairs$positive==score.pairs$negative)))/nrow(score.pairs)
  return(auroc)
}
```

```
#5-fold cross-validated lasso
#### CV Lasso Function for NLP Outcomes####
cvlasso_depNLP <- function(ytrain, Xtrain, ydev, Xdev){
  ptm <- proc.time() #Start execution timer
  #Storage for predictions, auc
  preds_cv <- actuals_cv <- preds <- actuals <- auc <- lambdamins <- rsq <- list()
  for(i in 1:5){
    indices_cv <- sample(1:5, length(ydev), replace=TRUE)
    ydevtest_cv <- ydev[indices_cv == i]
    Xdevtest_cv <- Xdev[indices_cv == i, ]
    ydevtrain_cv <- ydev[indices_cv != i]
    Xdevtrain_cv <- Xdev[indices_cv != i, ]
    #Cross validating five fold glmnet to pick optimal lambda value
    lasso_cv <- cv.glmnet(x=Xdevtrain_cv, y=ydevtrain_cv, alpha=1,
                          type.measure = "deviance", nfolds=5)
    #Fit model using best lambda from cross validation
    lasso <- glmnet(x=Xdevtrain_cv, y=ydevtrain_cv, alpha=1,
                    lambda=lasso_cv$lambda.min)
    #Predict on test data with fitted, trained model
    preds_cv1 <- predict(lasso, newx = Xdevtest_cv)
    auc[i] <- AUROC_depNLP(preds_cv1, ydevtest_cv)
    lambdamins[i] <- lasso_cv$lambda.min

    #Store all predictions and actual values in vectors
    for(j in 1:length(preds_cv1)){
      preds_cv <- unlist(rbind(preds_cv, preds_cv1[[j]]))
      actuals_cv <- unlist(rbind(actuals_cv, ydevtest_cv[[j]]))
    }
  }
  aucalltrain <- c()
  lambdaminall <- c()
  for(k in 1:5){
    aucalltrain <- rbind(aucalltrain, auc[[k]])
    lambdaminall <- rbind(lambdaminall, lambdamins[[k]])
  }
  #Pick best lambda by maximizing the AUROC
  lambda_auc <- cbind(lambdaminall, aucalltrain)
  colnames(lambda_auc) <- c("Lambda", "AUROC")
  lambda_aucdf <- data.frame(lambda_auc)
  lambda_best1 <- lambda_aucdf[lambda_aucdf$AUROC ==
                                 max(lambda_aucdf[,"AUROC"]),
                               "Lambda"]
  lambda_best <- ifelse(length(lambda_best1) > 1, min(lambda_best1), lambda_best1)
  auctrainmean <- mean(aucalltrain)
  auctrain1 <- AUROC_depNLP(preds_cv, actuals_cv)
  #Fit model using best lambda from earlier cross validation
  lassofinal <- glmnet(x=Xtrain, y=ytrain, alpha=1,
                       lambda=lambda_best, standardize = FALSE)
```

```r
  #Pull betas from model and present with matched covariate names
  betas <- lassofinal$beta
  nonzero_betas <- cbind(row.names(betas)[which(betas[,1] != 0)],
                         betas[which(betas[,1] != 0)])
  runtime <- proc.time() - ptm
  return(list("Nonzero Betas" = nonzero_betas,
              "Betas" = betas,
              "Total Nonzero Betas" = nrow(nonzero_betas),
              "Lambda Best" = lambda_best,
              "Mean Train AUROC" = auctrainmean,
              "Development AUROC" = auctrain1,
              "Runtime" = runtime,
              "Trained Model" = lassofinal))
}
```

```r
#Selective Inference performed using results of lasso regression
SIlasso <- function(lasso_results, xtrain, ytrain){
  trained_model_lasso <- lasso_results$`Trained Model`
  lambda_best <- lasso_results$`Lambda Best`
  trainauc <- lasso_results$`Mean Train AUROC`
  n_train <- length(ytrain)
  betas <- coef(trained_model_lasso, x=xtrain, y=ytrain, s=lambda_best, exact=TRUE)[-1]
  intercept <- coef(trained_model_lasso, x=xtrain, y=ytrain, s=lambda_best, exact=TRUE)[1]
  out_sample <- fixedLassoInf(xtrain, ytrain, betas, lambda_best)
  compare_coefs <- data.frame(cbind(lasso_results$`Nonzero Betas`,
                                    out_sample$coef0, out_sample$pv, out_sample$ci))
  colnames(compare_coefs) <- c('Variable', 'Nonzero Betas OG Lasso',
                               'SI Lasso Betas', 'SI p-value', 'Lower CI', 'Upper CI')
  return(list("Compare_Coefficients" = compare_coefs,
              "intercept" = intercept,
              "Train/Dev AUROC" = trainauc))
}
```

```r
#Pull out the SI betas that result from SIlasso function
linear_SI <- function(coefdf, intercept, xtestdf, ytest){
  sumpred <- predSI <- c()
  for(i in 1:nrow(xtestdf)){
    for(j in 1:nrow(coefdf)){
      sumpred <- rbind(sumpred,
                       xtestdf[noquote(coefdf$Variable)[j]][i,]
                       *as.numeric(coefdf$`SI Lasso Betas`[[j]]))
    }
    colpred <- matrix(sumpred, ncol=i)
    predSI <- rbind(predSI, (intercept + sum(colpred[,i])))
  }
  SIlassoaurocfinal <- AUROC_depNLP(predSI, matrix(ytest, ncol=1))
  dfpreds <- data.frame(cbind(matrix(ytest, ncol=1), predSI))
  colnames(dfpreds) <- list('Actuals', 'Predictions')
  rsq <- cor(ytest, predSI)^2
  return(list("DF Predictions" = dfpreds,
              "AUROC" = SIlassoaurocfinal,
              "r-square" = rsq))
}
```

Set up for stepwise process for interpreting the contribution of each variable type

```
##Importing dataframe and isolating train-test-dev, outcome, and covariates
dat_NLP_all_30 <- read.csv("/Users/SMR232/Documents/R21/code/30days_NLP_plus_all.csv")

y_all_NLP_30 <- dat_NLP_all_30$GroupedEPDSAverage

namesNLP <- colnames(dat_NLP_all_30)
datv11NLP <- dat_NLP_all_30[ ,!duplicated(namesNLP)]

traintestdev <- datv11NLP[, "traintestdev"]
datv1NLP <- subset(datv11NLP, select = -c(traintestdev,GroupedEPDSAverage))
datv1NLP <- apply(datv1NLP, 2,
                  function(x) as.numeric(as.character(x))) #Make sure in numeric format
```

Example of single variable type model (i.e., model #'s 1-4)

```
####Setting up for model #1--remove personal history, mood,
#and pregnancy-specific symptoms
datv1NLP_justNLP <-
  subset(datv1NLP,
         select= -c(AgeAtStartDate, WhiteCaucasian, ##Personal history
                    HispanicLatinx, Asian, BlackOrAfricanAmerican,
                    IncomeBinary, CollegeOrMore,
                    Depression, Anxiety,
                    MaxMood, MinMood, AvgMood, ##Mood
                    CriticalSymptom, ##Pregnancy-specific symptoms
                    SignificantSymptom, CommonSymptom,
                    SelfEnteredSymptom))
dat_development_NLP_30_justNLP <-
  matrix(datv1NLP_justNLP[which(traintestdev == 'dev'),],
                                        ncol=ncol(datv1NLP_justNLP))
colnames(dat_development_NLP_30_justNLP) <- colnames(datv1NLP_justNLP)

dat_training_NLP_30_justNLP <-
  matrix(datv1NLP_justNLP[which(traintestdev == 'train'),],
                                      ncol=ncol(datv1NLP_justNLP))
colnames(dat_training_NLP_30_justNLP) <- colnames(datv1NLP_justNLP)

dat_test_NLP_30_justNLP <-
  data.frame(matrix(datv1NLP_justNLP[which(traintestdev == 'test'),],
                                          ncol=ncol(datv1NLP_justNLP)))
colnames(dat_test_NLP_30_justNLP) <- colnames(datv1NLP_justNLP)
```

Example of variable type pairs model (i.e., model #'s 5-10)

```
####Setting up for model #10--select only mood + pregnancy-specific symptoms
####Mood + Symptoms####
datv1NLP_moodsymptoms <- subset(datv1NLP,
                                select = c(MinMood, MaxMood, AvgMood,
                                           CriticalSymptom, SignificantSymptom,
                                           CommonSymptom, SelfEnteredSymptom))
dat_development_NLP_30_moodsymptoms <-
```

```
  matrix(datv1NLP_moodsymptoms[which(traintestdev == 'dev'),],
                                              ncol=ncol(datv1NLP_moodsymptoms))
colnames(dat_development_NLP_30_moodsymptoms) <- colnames(datv1NLP_moodsymptoms)

dat_training_NLP_30_moodsymptoms <-
  matrix(datv1NLP_moodsymptoms[which(traintestdev == 'train'),],
                                           ncol=ncol(datv1NLP_moodsymptoms))
colnames(dat_training_NLP_30_moodsymptoms) <- colnames(datv1NLP_moodsymptoms)

dat_test_NLP_30_moodsymptoms <-
  data.frame(matrix(datv1NLP_moodsymptoms[which(traintestdev == 'test'),],
                                                  ncol=ncol(datv1NLP_moodsymptoms)))
colnames(dat_test_NLP_30_moodsymptoms) <- colnames(datv1NLP_moodsymptoms)
```

Example of groups of 3 variable types model (i.e., model #'s 11-14)

```
####Setting up for model #12--remove mood only
####NLP + Personal History + Symptoms####
datv1NLP_demossymptomsNLP <- subset(datv1NLP, select = -c(MinMood, MaxMood, AvgMood))
dat_development_NLP_30_demossymptomsNLP <-
  matrix(datv1NLP_demossymptomsNLP[which(traintestdev == 'dev'),],
         ncol=ncol(datv1NLP_demossymptomsNLP))
colnames(dat_development_NLP_30_demossymptomsNLP) <- colnames(datv1NLP_demossymptomsNLP)

dat_training_NLP_30_demossymptomsNLP <-
  matrix(datv1NLP_demossymptomsNLP[which(traintestdev == 'train'),],
         ncol=ncol(datv1NLP_demossymptomsNLP))
colnames(dat_training_NLP_30_demossymptomsNLP) <- colnames(datv1NLP_demossymptomsNLP)

dat_test_NLP_30_demossymptomsNLP <-
  data.frame(matrix(datv1NLP_demossymptomsNLP[which(traintestdev == 'test'),],
                    ncol=ncol(datv1NLP_demossymptomsNLP)))
colnames(dat_test_NLP_30_demossymptomsNLP) <- colnames(datv1NLP_demossymptomsNLP)
```

All variable types model (i.e., model #15), as well as outcome for all models

```
####Setting up for model #15--All variable types
####NLP + Personal History + Symptoms + Mood ####
dat_development_NLP_30_all <- matrix(datv1NLP[which(traintestdev == 'dev'),],
                                     ncol=ncol(datv1NLP))
colnames(dat_development_NLP_30_all) <- colnames(datv1NLP)

dat_training_NLP_30_all <- matrix(datv1NLP[which(traintestdev == 'train'),],
                                  ncol=ncol(datv1NLP))
colnames(dat_training_NLP_30_all) <- colnames(datv1NLP)

dat_test_NLP_30_all <-
  data.frame(matrix(datv1NLP[which(traintestdev == 'test'),],
                                         ncol=ncol(datv1NLP)))
colnames(dat_test_NLP_30_all) <- colnames(datv1NLP)

y_development_NLP_30_all <- y_all_NLP_30[which(traintestdev == 'dev')]
```

```r
y_training_NLP_30_all <- y_all_NLP_30[which(traintestdev == 'train')]

y_test_NLP_30_all <- y_all_NLP_30[which(traintestdev == 'test')]
```

For the rest of this document, we will use model #15 as the example.

```r
####Model #15: NLP + Personal History + Symptoms + Mood ####
####Lasso training/cross-validation using train + development
lasso30dayNLP_all <- cvlasso_depNLP(y_training_NLP_30_all,
                                    dat_training_NLP_30_all,
                                    y_development_NLP_30_all,
                                    dat_development_NLP_30_all)

####SI lasso adjustments on train + development
lasso30dayNLP_all_SI <- SIlasso(lasso30dayNLP_all,
                                dat_training_NLP_30_all,
                                y_training_NLP_30_all)

####Predicting on test set
results_SI_test30_demosmoodsymptomsNLP <-
  linear_SI(lasso30dayNLP_all_SI$Compare_Coefficients,
            lasso30dayNLP_all_SI$intercept,
            dat_test_NLP_30_all,
            y_test_NLP_30_all)

####Bootstrapped confidence interval
testdemosmoodsymptomsNLP30day_bin <-
  ifelse(results_SI_test30_demosmoodsymptomsNLP$`DF Predictions`$Actuals >= 14, 1, 0)
ci.auc(testdemosmoodsymptomsNLP30day_bin,
       results_SI_test30_demosmoodsymptomsNLP$`DF Predictions`$Predictions,
       method='bootstrap')
```
