# Supplementary material for: Digital phenotyping of depression during pregnancy using self-report data
Source: J Affect Disord. Author manuscript; Available in PMC 2024 Nov 17. (PMC11569620; doi:10.1016/j.jad.2024.08.029)
Supplement: Supplementary Material [file NIHMS2034961-supplement-Supplementary_Material.zip › 1-s2.0-S0165032724012229-mmc4.docx]

**Supplemental Table 2. The additive effect of feature types in predicting an EPDS score in a 60-day timeframe.**

| # | Model Description | No. of Coefficients Selected/No. of Covariates | Test Set AUROC [CI] | Test Set R^2^ |
| --- | --- | --- | --- | --- |
| 1 | Language features | 4/429 | .70 [.57, .81] | .15 |
| 2 | Personal history | 5/9 | .68 [.56, .79] | .10 |
| 3 | Mood | 1/3 | .76 [.63, .86] | .16 |
| 4 | Pregnancy-related symptoms | 2/4 | .73 [.61, .84] | .07 |
| 5 | Personal history  + Language features | 5/438 | .68 [.56, .79] | .10 |
| 6 | Mood  + Language features | 1/432 | .76 [.64, .87] | .19 |
| 7 | Pregnancy-related symptoms  + Language features | 9/433 | .72 [.60, .83] | .12 |
| 8 | Personal history  + Mood | 6/12 | .82 [.72, .90] | .27 |
| 9 | Personal history  + Pregnancy-related symptoms | 7/13 | .74 [.64, .83] | .13 |
| 10 | Mood  + Pregnancy-related symptoms | 3/7 | .80 [.69, .90] | .21 |
| 11 | Personal history + Mood  + Language features | 3/441 | .78 [.67, .89] | .23 |
| 12 | Personal history  + Pregnancy-related symptoms + Language features | 4/442 | .64 [.51, .76] | .07 |
| 13 | Mood  + Pregnancy-related symptoms + Language features | 1/436 | .76 [.64, .87] | .19 |
| 14 | Personal history  + Mood  + Pregnancy-related symptoms | 7/16 | .83 [.74, .91] | .36 |
| 15 | Personal history  + Mood  + Pregnancy-related symptoms + Language features | 5/445 | .80 [.70, .89] | .25 |
