# Supplementary material for: Digital phenotyping of depression during pregnancy using self-report data
Source: J Affect Disord. Author manuscript; Available in PMC 2024 Nov 17. (PMC11569620; doi:10.1016/j.jad.2024.08.029)
Supplement: Supplementary Material [file NIHMS2034961-supplement-Supplementary_Material.zip › 1-s2.0-S0165032724012229-mmc2.docx]

**Supplemental Figure 1. Features and significance level selected by top-performing 30-day models.**


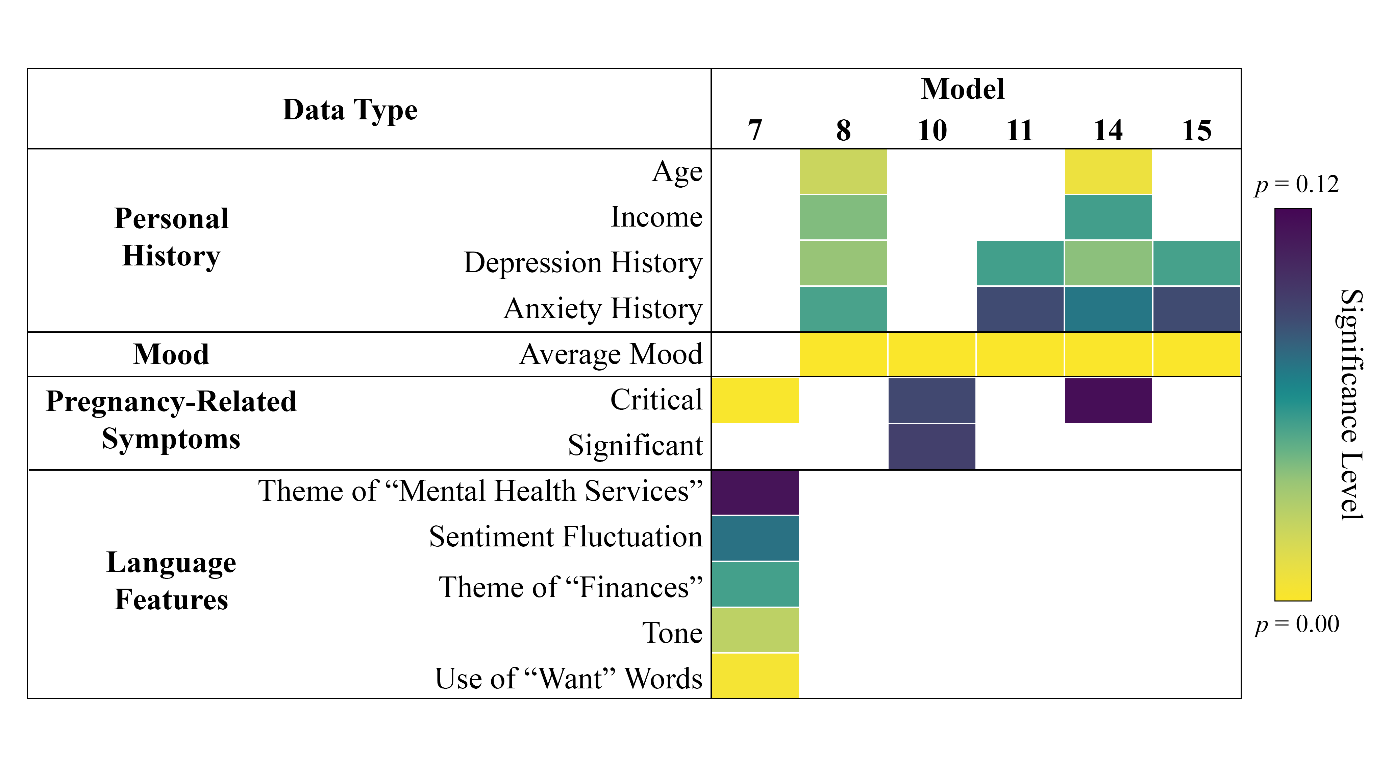
*Note: all models included in this table achieve an AUROC of at least 0.80. Lack of color for a data type indicates that the variable was not included in the model.*
